# Regression of devil facial tumour disease following immunotherapy in immunised Tasmanian devils

**DOI:** 10.1038/srep43827

**Published:** 2017-03-09

**Authors:** Cesar Tovar, Ruth J. Pye, Alexandre Kreiss, Yuanyuan Cheng, Gabriella K. Brown, Jocelyn Darby, Roslyn C. Malley, Hannah V. T. Siddle, Karsten Skjødt, Jim Kaufman, Anabel Silva, Adriana Baz Morelli, Anthony T. Papenfuss, Lynn M. Corcoran, James M. Murphy, Martin J. Pearse, Katherine Belov, A. Bruce Lyons, Gregory M. Woods

**Affiliations:** 1 Menzies Institute for Medical Research, University of Tasmania, Hobart, Tasmania 7000, Australia; 2 Faculty of Veterinary Science, University of Sydney, Sydney, New South Wales 2006, Australia; 3 Department of Health and Human Services, Hobart, Tasmania 7000, Australia; 4 School of Medicine, University of Tasmania, Hobart, Tasmania 7000, Australia; 5 Royal Hobart Hospital, Hobart, Tasmania 7000, Australia; 6 Centre for Biological Sciences, University of Southampton, Southampton SO17 1BJ, United Kingdom; 7 Department of Cancer and Inflammation, University of Southern Denmark, 5000 Odense C, Denmark; 8 Department of Pathology, University of Cambridge, Cambridge CB2 1QP, United Kingdom; 9 CSL Ltd., Bio21 Institute, Melbourne, Victoria 3010, Australia; 10 The Walter and Eliza Hall Institute of Medical Research, Parkville, Victoria 3052, Australia; 11 Department of Medical Biology, The University of Melbourne, Parkville, Victoria 3010, Australia

## Abstract

Devil facial tumour disease (DFTD) is a transmissible cancer devastating the Tasmanian devil (*Sarcophilus harrisii*) population. The cancer cell is the ‘infectious’ agent transmitted as an allograft by biting. Animals usually die within a few months with no evidence of antibody or immune cell responses against the DFTD allograft. This lack of anti-tumour immunity is attributed to an absence of cell surface major histocompatibility complex (MHC)-I molecule expression. While the endangerment of the devil population precludes experimentation on large experimental groups, those examined in our study indicated that immunisation and immunotherapy with DFTD cells expressing surface MHC-I corresponded with effective anti-tumour responses. Tumour engraftment did not occur in one of the five immunised Tasmanian devils, and regression followed therapy of experimentally induced DFTD tumours in three Tasmanian devils. Regression correlated with immune cell infiltration and antibody responses against DFTD cells. These data support the concept that immunisation of devils with DFTD cancer cells can successfully induce humoral responses against DFTD and trigger immune-mediated regression of established tumours. Our findings support the feasibility of a protective DFTD vaccine and ultimately the preservation of the species.

Devil facial tumour disease (DFTD) is a transmissible Schwann cell cancer^1^ that has decimated the Tasmanian devil (*Sarcophilus harrisii*) population^2^. The tumour is identified by its morphology and expression of the myelin-associated protein, periaxin^3^. Since its recognition in the far northeast of Tasmania in 1996, the disease has spread over most of the devils’ natural range. It has been estimated that more than 80% of the devil population has been killed by DFTD^2^. Conservation efforts are in place to protect this species, including the establishment of an insurance population. The objective is to reintroduce a robust and genetically diverse population into the wild. This can only be achieved if these devils are protected from DFTD, hence the urgent need for a vaccine.

In DFTD, the cancer cell is the ‘infectious’ agent that is transmitted as an allograft by biting^2^. Tasmanian devils efficiently reject allogeneic skin grafts^4^ and are capable of producing both cytotoxic and antibody responses against xenogeneic cancer cells^5^. We have also shown that activation of devil mononuclear cells with mitogens and cytokines can induce cytotoxic responses against DFTD cells *in vitro*^6^. Recently, immune responses against DFTD cells were also detected in a small number of animals in the wild^7^. However, most devils with DFTD do not appear to produce an immune response against the allogeneic cancer cells^6^,^8^,^9^. The recognition of allografts and malignant cells by the adaptive immune system depends on the interaction of host T cells with molecules of the major histocompatibility complex class I (MHC-I) expressed on the surface of nucleated cells. As DFTD cancer cells do not express MHC-I, this is likely to be a key mechanism by which tumour cells escape immune detection in devils^10^. Exposure of DFTD cells with IFN-γ can restore MHC-I expression^10^, potentially leading to allo-recognition and forming the basis of a vaccine.

In this study we sought to establish whether induction of MHC-I expression on DFTD cells as components of immunisations and immunotherapy would correspond with anti-DFTD tumour responses. To further boost immune recognition, the immunisations with IFN-γ treated DFTD cells were incorporated with combinations of ISCOMATRIX™, Poly I:C and CpG. These adjuvants promote innate, humoral and cell mediated anti-tumour immunity^11^. This paper illustrates the potential for vaccination to induce humoral responses and, with additional immunotherapy, trigger DFTD immune-mediated regression *in vivo.*

## Results

The aim of the current study was to explore immunisation protocols to enhance protective responses against DFTD. These included immunisation with DFTD cells expressing MHC-I to increase their antigenicity, in combination with ISCOMATRIX™, Poly I:C and CpG, adjuvants that enhance both innate and adaptive immune responses. As access to Tasmanian devils for research purposes is limited, we were restricted to the use of nine healthy devils, some of which had reached an advanced age. These devils were used over a five-year period. In an endeavour to improve the immune response, four immunisation protocols were tested sequentially. Seven devils were immunised with a variety of cell preparations and adjuvants to determine if they could be protected against the development of DFTD following challenge with live DFTD cells. One devil was used as an adjuvant-alone control and one devil as a non-immunised control. Devils were monitored for adverse reactions to the immunisations and none were identified. In the absence of a reliable cytotoxicity assay we used DFTD-specific IgG antibody levels in devil serum to evaluate the immune responses after the immunisations in each protocol. As devils were immunised with either MHC-I^+^ or MHC-I^−^ DFTD cells, antibody reactivity was evaluated against cytokine treated (MHC-I^+^) and untreated (MHC-I^−^) DFTD cells. As IgG production requires T cell help, this also provided an indicator of T cell involvement. Six of the immunised devils and the non-immunised control devil were later challenged with live DFTD cells. If tumours developed, immunotherapy was commenced and tumour rejection and anti-tumour antibody responses were measured. Tumour biopsies were taken before and after immunotherapy, and evaluated for immune cell infiltration by immunohistochemistry. An outline of the immunisations, challenges and tumour development is presented in Supplementary Table S1.

**Figure 1:**
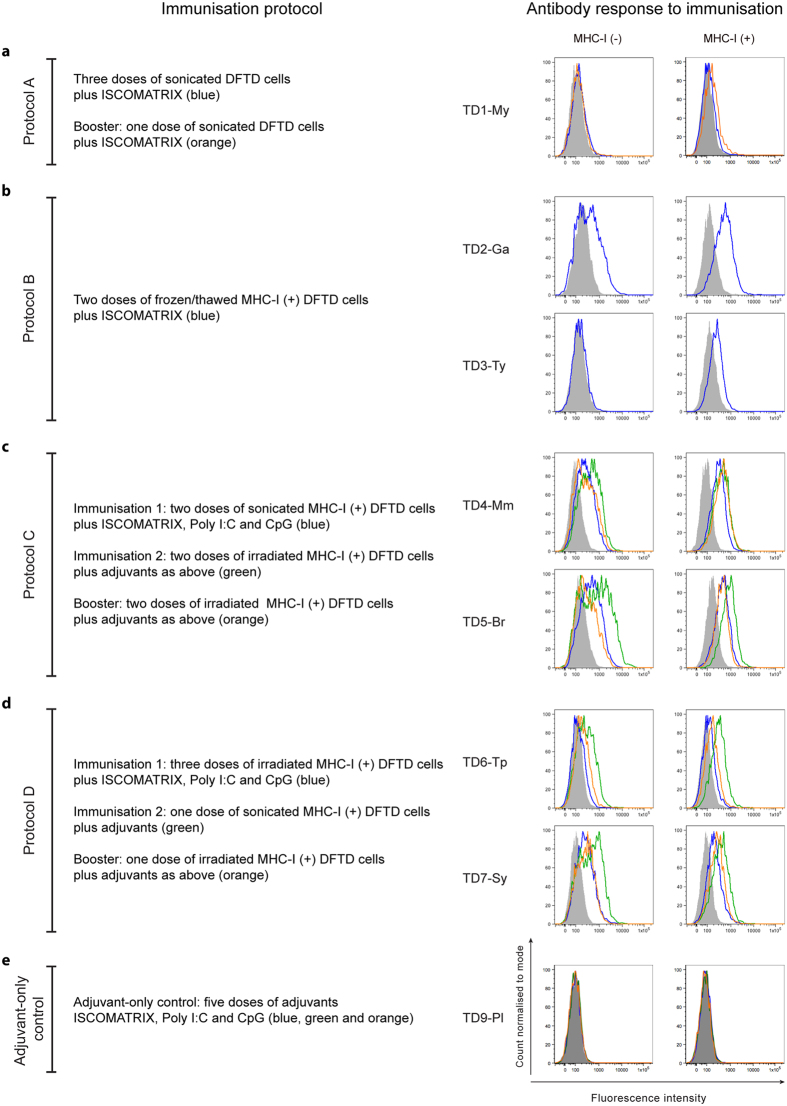
Antibody responses to immunisation in protocols A to D and adjuvant control. Immunisation with MHC-I^+^ DFTD cells consistently induced antibodies responses. The left column shows a brief description of the immunisation protocols and the histograms in the right column show the corresponding antibody responses against both MHC-I^+^ and MHC-I^−^ DFTD cells assessed by flow cytometry. Each colour in the histogram relates to a particular protocol as indicated. (**a**) Protocol A (TD1-My), immunisation with DFTD cell protein extracts did not induce antibody responses. (**b**) Protocol B, immunisation with MHC-I^+^ DFTD cells elicited antibodies responses particularly against MHC-I^+^ cells in both TD2-Ga and TD3-Ty devils. Immunisation with a combination of firstly sonicated and then irradiated MHC-I^+^ DFTD cells in Protocol C (**c**) or firstly irradiated and then sonicated in Protocol D (**d**) consistently induced antibody responses in the four devils. (**e**) Immunisation of the adjuvant control devil TD9-Pl followed the same regime as Protocol D but received only the adjuvant component of the vaccine. This devil did not produce detectable antibodies against DFTD cells.

**Figure 2:**
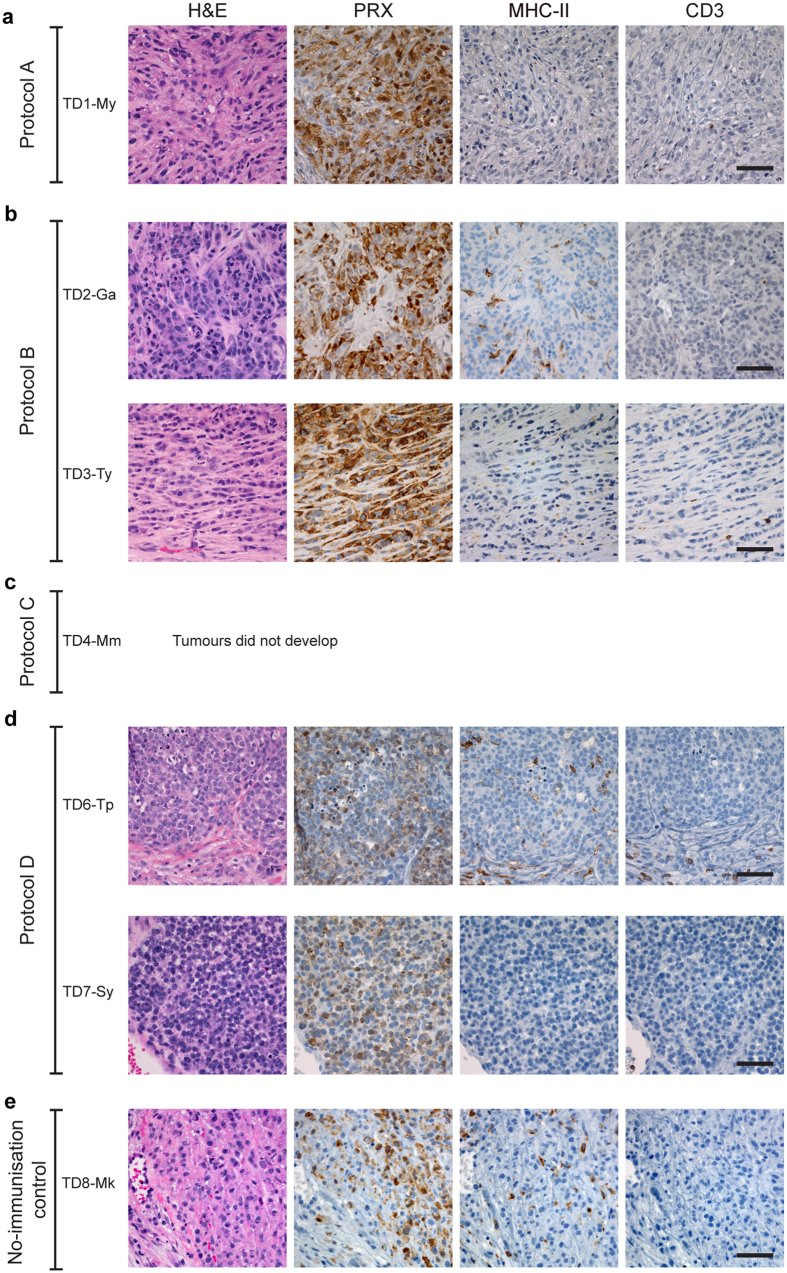
Histology and immunohistochemistry of DFTD tumours following challenge, showing lack of immune cell infiltration. (**a**) Sections of a biopsy of the induced tumour in TD1-My (Immunisation Protocol A) taken 10 weeks after challenge. There is no evidence of immune cell infiltration. (**b**) Development of grafted DFTD tumours in devils immunised with Protocol B. The biopsy sections of the tumour in TD2-Ga taken 12 weeks after challenge show scattered MHC-II^+^ cells but virtually no infiltrating CD3^+^ cells. Similarly, no immune cell infiltration was detected in a biopsy of the DFTD tumour in TD3-Ty taken 10 weeks after the challenge. (**c**) TD4-Mm (Protocol C) did not develop tumours after the challenge with live DFTD cells. (**d**) Development of grafted DFTD tumours in devils immunised with Protocol D. TD6-Tp developed DFTD tumours after challenge at both sides of injection (left hand side - LHS, and right hand side - RHS of the rump). The images are representative histology of a biopsy from the LHS tumour taken 14 weeks after challenge showing very poor immune cell infiltration. TD7-Sy also developed DFTD tumours at both sides of injection. The images show no evidence of immune cell infiltration in a biopsy of the LHS tumour taken 20 weeks after challenge. (**e**) The adjuvant control (TD8-Mk) developed tumours at both sides of the challenge. A biopsy of the LHS tumour taken 10 weeks after challenge shows scattered MHC-II^+^ cells within the tumour and very occasional CD3^+^ cells. All panels (**a** to **e**) standard haematoxylin and eosin (H&E) staining and immunohistochemical labelling using anti-periaxin (PRX) antibody to detect DFTD tumour cells and anti-MHC-II and anti-CD3 antibodies to detect an immune response. Scale bar, 50 μm

**Figure 3:**
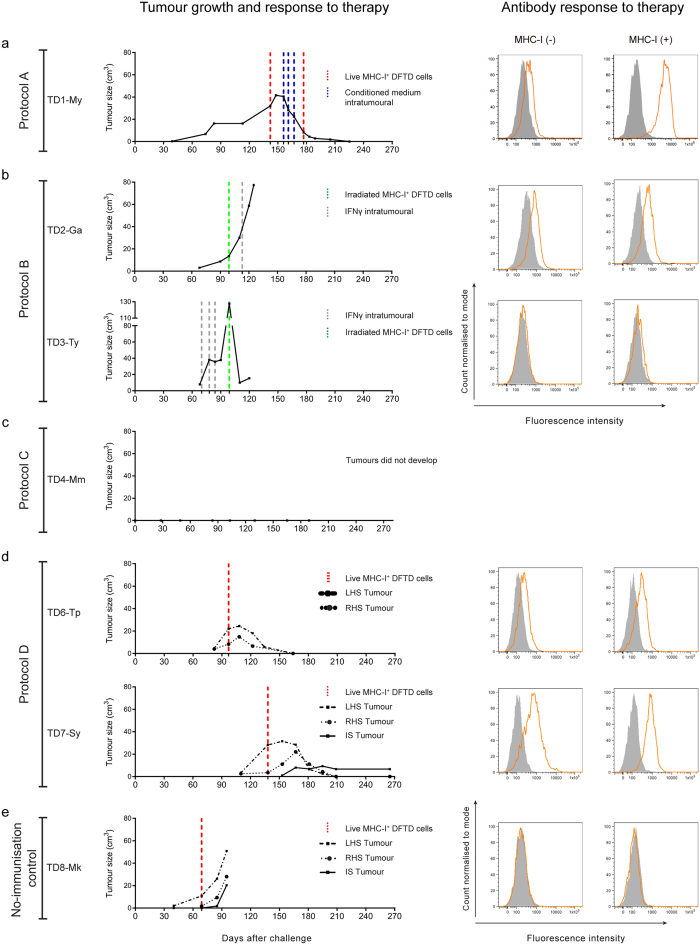
Tumour growth and antibody responses following immunotherapy. Tumour regression associated with antibody responses was observed in three devils after therapy with live MHC-I^+^. Graphs on the left show a time-line of the growth of the induced tumours following challenge with live DFTD cells (day 0) for each protocol (**a** to **d**) and the no-immunisation control (**e**). Immunotherapy protocols are indicated in coloured vertical dashed lines. Histograms on the right show the antibody responses after the therapy assessed by flow cytometry against both MHC-I^−^ and MHC-I^+^ DFTD cells. (**a**) Protocol A, immunotherapy in TD1-My induced complete tumour regression and was associated with high levels of antibody, particularly against MHC-I^+^ DFTD cells. (**b**) Protocol B, immunotherapy in TD2-Ga was ineffective and the tumour grew. Antibodies were detected against both MHC-I^−^ and MHC-I^+^ DFTD cells. Immunotherapy in TD3-Ty was ineffective in controlling tumour growth. The tumour ulcerated and dislodged from the skin. A few weeks later the tumour re-established and continued to increase in size. Antibodies were not detected in the serum. (**c**) Protocol C. TD4-Mm did not develop tumours after challenge. This devil died 189 days after challenge. A post-mortem examination did not detect DFTD tumours or metastases. (**d**) Protocol D. Immunotherapy in TD6-Tp consisting of a single injection of live MHC-I^+^ DFTD cells given in the interscapular region induced complete regression of both tumours. The same immunotherapy in TD7-Sy also induced complete regression of both tumours. A small third tumour developed in the site of the immunotherapy injection. This tumour did not increase in volume and immune cell infiltration was observed in histology (see Supplementary Table S2). Both devils TD6-Tp and TD7-Sy had elevated serum antibodies against DFTD cells. (**e**) The therapy administered to the no-immunisation control (TD8-Mk) was ineffective and both tumours grew. A third tumour developed in the site of the immunotherapy and this tumour also continued to increase in size. Antibodies were not detected after therapy.

**Figure 4:**
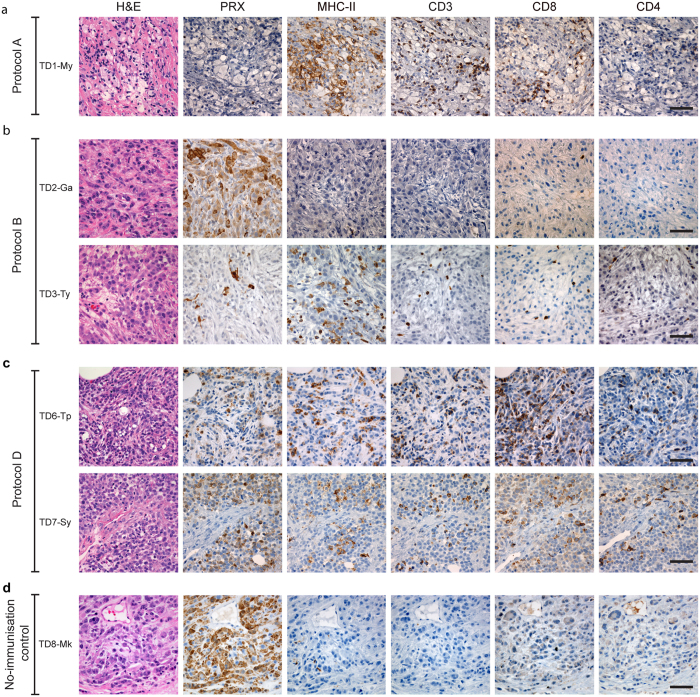
Tumour histology and immunohistochemistry of DFTD tumours following immunotherapy showing tumour regression and immune cell infiltration. (**a**) Protocol A. Biopsy sections of the tumour site of TD1-My taken one week after completion of immunotherapy. Tumour regression correlated with strong immune cell infiltration of MHC-II^+^ cells and T cells, mainly CD8^+^ cells. (**b**) Protocol B. A tumour biopsy taken one week after the last therapy in TD2-Ga shows no evidence of immune cell infiltration. Biopsies of the scar tissue 3 weeks after dislodgment of the tumour in TD3-Ty show the persistence of few DFTD (PRX^+^) cells and a large number of MHC-II^+^ cells but very few CD3^+^ cells. (**c**) Protocol D. Tumour regression in TD6-Tp and TD7-Sy correlated with strong immune cell infiltration of MHC-II^+^ cells and CD3^+^ cells with CD8^+^ cells more abundant than CD4^+^ cells as evidenced by biopsies taken 4 weeks after therapy. (**d**) No-immunisation control (TD8-Mk), a biopsy of the LHS tumour taken 4 weeks after therapy shows well established DFTD tumours with virtually no immune cell infiltration. Standard haematoxylin and eosin (H&E) staining and immunohistochemical labelling using anti-periaxin (PRX) antibody and anti-MHC-II, CD3, CD8 and CD4 antibodies. Scale bar, 50 μm.

A comparison of the four protocols can be seen in Figs 1–4. The responses to immunisation are shown in Fig. 1 and highlight that immunisation with MHC-I^+^ DFTD cells consistently produced antibody responses. Figure 2 shows the histology of the engrafted DFTD tumours, which occurred in six of the seven devils challenged with live tumour cells. Immunohistochemistry did not reveal evidence of immune cell infiltration into the tumours. Figure 3 outlines the growth of the engrafted tumours and their response to immunotherapy. Immunotherapy with live MHC-I^+^ DFTD cells was associated with tumour regression in three devils and the regression correlated with antibody responses against DFTD cells. The histology in Fig. 4 supports the immune-mediated regression of the tumours as indicated by the large numbers of MHC-II and CD3 positive cells infiltrating the tumour.

The following sections describe the assessment of the responses to immunisation and immunotherapy in each protocol.

### Protocol A

Devil TD1-My was immunised subcutaneously three times with protein extracted from heat-treated DFTD cells and boosted six months after the third immunisation. Each immunisation and the booster used the ISCOMATRIX™ adjuvant. This devil did not show any evidence for an antibody response against the DFTD cells (Fig. 1a). While there was no DFTD-specific antibody response detectable in the *in vitro* assay, an *in vivo* immune response may have occurred. To test this, the devil was challenged with 25,000 live DFTD cells one month after the booster immunisation in one site in the rump. A tumour was detected 37 days after this challenge. DFTD was confirmed on biopsy by periaxin expression and there were no signs of immune cell infiltration in the tumour (Fig. 2a).

The lack of an antibody response indicated that the DFTD cells were poorly recognised by this devil’s immune system. When the tumour in this animal reached approximately 30 cm^3^ and with no signs of regression, the devil was injected subcutaneously with live interferon-γ (IFN-γ) treated MHC-I^+^ DFTD cells between the shoulder blades. As DFTD cells cultured with IFN-γ upregulate MHC-I expression^9^, they have the potential to become targets for an allogeneic immune response. The original tumour continued to increase in size, but after one week it began to regress (Fig. 3a). To maintain the response, a cytokine rich conditioned medium (supernatant obtained from mitogen stimulated devil lymphocytes) was injected intra-tumourally each week for three weeks. This was followed by an additional injection of live MHC-I^+^ DFTD cells near the tumour. The tumour continued to regress until it was no longer palpable four weeks after the last immunotherapy (Fig. 3a).

One week after all treatments were completed, the serum contained elevated levels of antibodies against MHC-I^+^ DFTD cells, almost 30 times the median fluorescence intensity (MFI) of the pre-immune serum. Antibodies against MHC-I^−^ DFTD cells were also detected, but at lower levels (Fig. 3a). A tumour biopsy, taken a week after regression was first detected, showed sparse DFTD cells, with a strong infiltration of MHC-II^+^ cells and CD3^+^ cells (predominantly CD8^+^) into the tumour (Fig. 4a, Supplementary Table S2). Table 1 describes the immunotherapy and summarises the immune response to the therapy.

Due to an age-related health problem, this devil was euthanised 40 weeks after the last treatment. There were no signs of tumour recurrence or metastases during post-mortem examination. The remarkable T cell infiltration into the tumour and the strong antibody response provided the first evidence that immunotherapy can stimulate the devil’s immune system to recognise and target an established DFTD tumour. One concern was that immunotherapy with live MHC-I^+^ DFTD cells could pose a risk of tumour engraftment at the tumour immunotherapy site. Therefore, when immunisation protocols failed to protect against experimentally induced DFTD the subsequent immunotherapy was inoculation with irradiated IFN-γ treated MHC-I^+^ DFTD cells (to mimic intact live MHC-I^+^ DFTD cells) and IFN-γ therapy (protocol B).

### Protocol B

Two devils, TD2-GA and TD3-Ty were immunised with frozen/thawed DFTD cells that had been treated with either Trichostatin A (TSA), a histone deacetylase inhibitor (TD2-Ga) or cytokine rich conditioned medium (TD3-Ty) to upregulate MHC-I expression. The adjuvant ISCOMATRIX™ was used in all immunisations.

TD2-Ga developed low to medium antibody responses against IFN-γ treated MHC-I^+^ DFTD cells and untreated DFTD cells (Fig. 1b). The devil was then challenged with 25,000 live DFTD cells and a DFTD tumour was first identified at the inoculation site 67 days after challenge. Immunohistochemistry at this time showed few MHC-II^+^ cells and occasional CD3^+^ cells, mostly located at the periphery of the tumours or in proximity to blood vessels. (Fig. 2b). Cells with dendritic morphology, presumably dendritic cells, were seen in the epidermis, dermis and subcutaneous tissue, but not associated with the tumours (Supplementary Table S2).

When the tumour reached approximately 20 cm^3^ in volume the devil was subcutaneously injected, on the rump near the tumour, with irradiated MHC-I^+^ DFTD cells followed one week later by an intra-tumoural injection of devil recombinant IFN-γ, which became available for the first time. The tumour continued to grow (Fig. 3b). For the duration of the immunotherapy, devil TD2-Ga maintained medium levels of antibodies against IFN-γ treated MHC-I^+^ and untreated DFTD cells (Fig. 3b). Tumour biopsies showed very few MHC-II^+^ cells and occasional T cells were present, but mostly in the surrounding connective tissue (Fig. 4b). This devil died naturally of an unrelated cause. A post-mortem showed an encapsulated DFTD tumour with strong evidence of tumour vascularisation including large blood vessels within the tumour. Few MHC-II^+^ and T cells were identified within the tumour. MHC-II^+^ and T cells were present in the granulation tissue and connective tissue away from the tumour. (Supplementary Table S2). Table 1 provides a summary of the immune responses to therapy.

TD3-Ty developed a low antibody response post-immunisation, but only against IFN-γ treated MHC-I^+^ DFTD cells (Fig. 1b). A DFTD tumour was first identified at the inoculation site 67 days after challenge with 25,000 live DFTD cells. Immunohistochemistry revealed almost no immune cell infiltration within the tumour (Fig. 2b). MHC-II^+^ and CD3^+^ cells were scarce, and when present were located at the periphery of the tumours or in proximity to blood vessels (Supplementary Table S2).

When the tumour reached approximately 20 cm^3^ in volume the devil received an intra-tumoural injection of IFN-γ each week for three weeks. The tumour continued to grow rapidly. Two weeks after the last dose of IFN-γ this devil was injected subcutaneously, on the rump near the tumour, with irradiated IFN-γ treated MHC-I^+^ DFTD cells (Fig. 3b). At this time the tumour appeared to ulcerate and it dislodged from the skin. A biopsy of the tumour site revealed granulation tissue, fibroblasts with an activated-like appearance and a small amount of necrotic tumour. Large dendritic MHC-II^+^ cells were identified in the periphery of the necrotic tumour cells associated with CD3^+^/CD8^+^ cells. Biopsies taken one, three and four weeks later showed the re-establishment of nests of DFTD tumours suggesting that dislodgment of the tumour may have been mechanical, and not due to an immune process (Supplementary Table S2). Serum antibodies against DFTD cells were not detected after treatment (Fig. 3b). MHC-II^+^ and CD3^+^ cells were present around the periphery of the tumour clusters but very few infiltrated the tumour (Fig. 4b). The tumour continued to grow and this devil was euthanised. A summary of the immunotherapy and the immune response to the therapy is described in Table 1.

From the immunisation of TD2-Ga and TD3-Ty, it was concluded that MHC-I^+^ DFTD cells induced better DFTD-specific IgG antibody responses. However, the immunisation protocol alone was not enough to induce an effective anti-tumour immune responses. Therefore, additional adjuvants were used in subsequent immunisation protocols.

### Protocol C

Two devils (TD4-Mm and TD5-Br) were immunised with sonicated DFTD cells that had been treated with IFN-γ to upregulate MHC-I (immunisation 1), followed by irradiated, IFN-γ treated DFTD cells (immunisation 2) and boosters with irradiated MHC-I^+^ DFTD cells. In all immunisations/boosters the adjuvants ISCOMATRIX™, Poly I:C and CpG were used.

TD4-Mm produced medium antibody responses against IFN-γ treated MHC-I^+^ and untreated DFTD cells (Fig. 1c). Two booster injections of irradiated MHC-I^+^ DFTD cells six and eleven months after the last immunisation did not appear to increase the antibody levels (Fig. 1c). This devil remained free of DFTD tumours for 189 days after challenge with live DFTD cells but was euthanized for age-related health reasons. Medium to high antibody responses against IFN-γ treated MHC-I^+^ DFTD cells and untreated DFTD cells were detected after the live tumour cell challenge and similar levels identified in the post-mortem sample (Table 1).

TD5-Br produced medium antibody responses against IFN-γ treated MHC-I^+^ and untreated DFTD cells (Fig. 1c). This devil was euthanized for age-related health reasons before it was challenged with live DFTD cells.

Immunisation Protocol C showed the first preliminary evidence that the combination of adjuvants led to a stronger anti-tumour antibody response. It is feasible that this strong immune response also prevented experimental DFTD tumour engraftment.

### Protocol D

Two devils (TD6-Tp and TD7-Sy) were immunised with irradiated DFTD cells that had been treated with IFN-γ to upregulate MHC-I (immunisation 1), followed by sonicated, IFN-γ treated MHC-I^+^ DFTD cells (immunisation 2) and a booster with irradiated MHC-I^+^ DFTD cells. All immunisations/boosters were in combination with ISCOMATRIX™, Poly I:C and CpG adjuvants.

TD6-Tp produced medium antibody responses against IFN-γ treated MHC-I^+^ DFTD cells and untreated DFTD cells, but only after receiving the second immunisation (Fig. 1d). 80 days following challenge with 25,000 live DFTD cells at the right hand side (RHS) of the rump and 100,000 live DFTD cells at the left hand side (LHS) of the rump, DFTD tumours developed at both sites. The higher dose was used to confirm that if a tumour did not develop, it was not due to insufficient cells being inoculated. Immunohistochemistry of the LHS tumour revealed only a few MHC-II^+^ and CD3^+^ cells mainly at the periphery of the tumour cell nests (Fig. 2d). When this tumour reached approximately 20 cm^3^ in volume the devil was subcutaneously injected in the interscapular region (between the shoulders blades), with live IFN-γ treated MHC-I^+^ DFTD cells. One week later the original tumours began to regress (Fig. 3d). Low to medium antibody responses against IFN-γ treated MHC-I^+^ and untreated DFTD cells were evident after immunotherapy (Fig. 3d). Biopsies of the LHS tumour, four weeks after immunotherapy, revealed moderate numbers of MHC-II^+^ cells towards the tumour periphery and a few within the tumour. A large number of CD3^+^ cells showed a similar distribution to the MHC-II^+^ cells, with CD8^+^ cells more abundant than CD4^+^ cells (Fig. 4c). Neither tumour (LHS and RHS) was palpable 70 days after immunotherapy commenced. Biopsies at the LHS tumour site showed scar tissue composed of hyalinised and relatively acellular dense connective tissue. Evidence of immune infiltration was indicated by moderate to large numbers of MHC-II^+^ cells, individually or in clusters, adjacent to the connective tissue. Similarly, a moderate to high number of CD3^+^ T cells paralleled the MHC-II^+^ cells (Supplementary Table S2).

TD7-Sy produced medium/high antibody responses against IFN-γ treated MHC-I^+^ DFTD cells and untreated DFTD cells after receiving immunisation 1. Responses increased after immunisation 2 (Fig. 1c). This devil was challenged with live DFTD cells at the RHS and LHS of the rump as described above and DFTD tumours developed at both inoculation sites 110 days later. Immunohistochemistry of the LHS tumour revealed limited immune cell infiltration (Fig. 2d). When the LHS tumour reached approximately 30 cm^3^ in volume the devil was subcutaneously injected, in the interscapular region, with live IFN-γ treated MHC-I^+^ DFTD cells. The tumours increased in size, but one week later both tumours began to regress (Fig. 3d). Medium to high antibody responses against IFN-γ treated MHC-I^+^ DFTD cells and untreated DFTD cells were evident after treatment (Fig. 3d). Biopsies of the LHS and RHS tumours, four weeks after immunotherapy, revealed moderate numbers of MHC-II^+^ cells towards the periphery of the tumour and some cells within the tumour. A large number of CD3^+^ cells showed a similar distribution to the MHC-II^+^ cells, with CD8^+^ cells more abundant than CD4^+^ cells (Fig. 4c). The LHS and RHS tumours were not palpable 70 days after treatment commenced.

With devil TD7-Sy, the live MHC^+^ DFTD cells that were used for immunotherapy developed into a small tumour (Fig. 3d). This tumour did not increase in volume after it had reached 10 cm^3^. MHC-II^+^ and CD3^+^ cells could be found throughout the tumour (Supplementary Table S2). A summary of the immunotherapy and the immune response to the therapy is described in Table 1.

Immunisation protocol D produced antibody responses to DFTD cells. Although this did not protect from tumour development, immunotherapy with a single injection of live MHC-I^+^ DFTD cells was followed by immune cell infiltration and tumour regression.

### Non-immunised control devil

TD8-Mk was not immunised and did not receive adjuvant. 40 days after challenge with live DFTD cells, tumours were palpable at both the 25,000 and 100,000 cell inoculation sites. The tumours continued to grow, with no indication of immune cell infiltration (Fig. 2e). When the tumour reached approximately 10 cm^3^ in volume the devil was subcutaneously injected, in the interscapular region, with live IFN-γ treated MHC-I^+^ DFTD cells. For this devil, the immunotherapy via injection of live MHC-I^+^ DFTD tumour cells produced a small tumour (Fig. 3e). There was no evidence for antibody production (Fig. 3e). Biopsies taken 28 days after the immunotherapy showed well established and encapsulated tumours. Average to large sized intra-tumoural blood vessels and extensive necrosis between the pockets of tumours were observed (Supplementary Table S2). A few foci of MHC-II^+^ cells appeared in the periphery of the tumours with very few within the tumour. Very few CD3^+^ (either CD4 and CD8) T cells were found within or surrounding the tumour (Fig. 4d). Due to the progression of tumour size, this devil was euthanised. Table 1 describes the immunotherapy and summarises the immune responses.

### Adjuvant control devil

TD9-Pl received five injections of the adjuvant components only (i.e. excluding DFTD cells) and did not produce detectable anti-DFTD antibodies (Fig. 1e). This devil was not challenged with live DFTD cells.

### MHC-I genotypes within the test cohort

Devils have three classical MHC-I genes, *Saha-UA, -UB* and *-UC*, with *Saha-UA* converted to a pseudogene in certain MHC haplotypes due to a 1.6-kb-long deletion in the genomic region^14^. All nine devils (seven immunised and two controls) and the DFTD tumour cell line were genotyped at these three loci for the peptide-binding domains (α1 and α2; Supplementary Table S3).

A total of 18 MHC-I variants was identified across all samples; 12 have been previously reported and assigned to MHC loci based on BAC contig assembly and sequencing and genotype analysis^14^,^15^, whereas six are novel variants (SahaI*35:02, 88:02, 99, 100, 101, and 102; GenBank accession numbers KY194692- KY194697). These were assigned to gene *UA, UB* or *UC* based on their phylogenetic relationship with previously reported variants (Supplementary Fig. S1). Four MHC-I variants, SahaI*35, 46, 90 and 28, were identified in the DFTD cells used in the immunisation and immunotherapy procedures. This result differs from the previously reported MHC genotype of the tumour (Pye *et al*. 2015), which is likely due to the use of different starting material. The previous study used tumour biopsies (Pye *et al*. 2015), which usually contain host connective tissue (Siddle *et al*.^10^ PNAS), whereas in this study tumour DNA was extracted from cell culture and therefore was unlikely to contain host DNA contamination. The other 14 MHC-I variants showed high sequence similarities (94.2% average amino acid sequence identity; Supplementary Table S4) with the four variants in the tumour, consistent with previous findings of low sequence variability in devil MHC genes^14^. Two control devils each shared one MHC-I antigen with DFTD cells (SahaI*35 in TD9-Pl and SahaI*28 in TD8-Mk), while five of the seven immunised devils, TD1-My (Protocol A), TD2-Ga and TD3-Ty (Protocol B), TD6-Tp and TD7-Sy (Protocol D), did not share any MHC-I molecules with the tumour. TD1-My (protocol A) had one MHC-I variant (SahaI*101) with relatively lower sequence similarity (89.2%) with one of the tumour antigen (SahaI*90). This animal produced lower antibody responses against MHC-I^+^ DFTD cells post-immunisation and post-booster than the other devils (Fig. 1). Of the two devils in protocol B, TD2-Ga appeared to produce stronger antibody responses than TD3-Ty. This may be attributed to TD2-Ga possessing MHC-I variant SahaI*87, which shows the lowest sequence identity among all variants to SahaI*46 (89.2%) and SahaI*28 (89.7%) of the tumour. The two devils in protocol C produced good immune responses even though they shared one and two MHC-I molecules with tumour cells. The two devils in protocol D, TD6-Tp and TD7-Sy, had similar MHC-I genotypes, both possessing variants SahaI*29, 36, 37 and 27. Both produced responses against DFTD cells.

## Discussion

Transmissible cancers rarely occur in nature and have only been recorded in domestic dogs, Tasmanian devils and shellfish^16^,^17^,^18^,^19^,^20^. Devil facial tumour disease has caused a devastating reduction in the wild devil population^21^. Intervention is required to prevent extinction of the wild population. Currently, healthy Tasmanian devils are kept in breeding programs as an insurance population with the goal of reintroducing healthy and genetically diverse devils into the wild. But releasing them into the wild will expose them to DFTD, hence the need for vaccination to protect them. For a vaccine to be effective it needs to induce an immune response against the cancer cells and show evidence of efficacy *in vivo*. Here we show the potential of immunisation strategies using DFTD cancer cells to induce immune responses against DFTD cells and regression of established tumours in devils.

Research on the Tasmanian devil is constrained due to the endangered status of the species. Despite this it was possible to test four immunisation protocols and to cautiously provide conclusions. For example, immunisation protocols C and D produced specific antibodies against MHC-I^+^ and MHC-I^−^ DFTD cells, thus immunisation with sonicated and irradiated MHC-I^+^ cells in the presence of multiple adjuvants promotes anti-DFTD responses. This immune response may have protected from DFTD development following challenge with live tumour cells. One immunised devil (TD4-Mm) did not develop DFTD and two immunised devils appeared to have a delayed onset of DFTD (TD6-Tp and TD7-Sy) compared to the single non-immunised control. Validation of these conclusions requires additional devils. This is particularly relevant for the non-immunised controls to more accurately determine time to tumour development and evidence for spontaneous regression, as occurs with experimental inoculation of dogs with canine transmissible venereal tumour (CTVT), the other transmissible tumour in a mammalian species^20^. Ethically it was undesirable to challenge additional devils knowing they would develop DFTD.

The development of DFTD tumours in devils immunised with DFTD cells and challenged with live DFTD cells, provided an opportunity to treat these devils with immunotherapy and evaluate an anti-tumour immune response *in vivo*. Our approach took advantage of the potential strong allogeneic responses induced by MHC incompatibilities. We injected devils with live DFTD cells that had been cultured *in vitro* with IFN-γ to upregulate MHC-I and three of the six devils with DFTD tumours underwent regression.

We recently provided evidence for tumour regression in a small proportion of wild devils with DFTD^7^. The presence of antibodies to DFTD cells in the wild devils that recovered provided evidence that regression was immune mediated. The identification of immune cell infiltration into the tumours undergoing regression in the experimental devils provides compelling evidence that DFTD tumours can be targeted and eliminated by the immune system.

Evaluation of the devils that showed tumour regression revealed three salient features (Table 2):

*Firstly*, regression correlated with prior immunisation with DFTD cells and there was a rise in antibody levels to DFTD cells following the immunotherapy. The priming effect appeared to be the combined action of the immunisation protocol and the adjuvants. ISCOMATRIX™ is a proprietary saponin based adjuvant, which is known to elicit a broad antibody response. This adjuvant induces the generation of antigen-specific CD8^+^ T cells and when combined with Toll-like receptor (TLR) agonists, causes the regression of established solid tumours^12^,^13^,^22^,^23^. We also used the TLR agonists CPG-1585 and CPG-2395, which stimulate via TLR9, and Poly I:C, which stimulates via TLR3. The combination of adjuvants in our vaccine is likely to engage multiple signalling pathways that support adaptive cellular immune responses in the Tasmanian devil.

*Secondly*, remission occurred in devils injected with live IFN-γ treated DFTD cells. Immunotherapy using live proliferating, but not irradiated, DFTD cells expressing surface MHC-I correlated with the cellular immune response observed at the tumour engraftment site. Proliferating DFTD tumour cells expressing MHC-I provide tumour antigens for presentation and the prior immunisation may allow presentation to memory cells, enabling more DFTD-specific T cells to be activated. We have recently shown that activated Tasmanian devil mononuclear cells can kill DFTD cells *in vitro*^6^.

*Thirdly*, remission correlated with T cell and MHC-II^+^ cell (potentially dendritic cell) infiltration into the DFTD tumour. The presence of tumour infiltrating lymphocytes, mostly CD3^+^ and CD8^+^, indicates an immunologically mediated regression. Indeed, after immunotherapy, we observed increased levels of IgG anti-DFTD antibodies in devils subjected to Protocols A and D. As IgG antibodies are T cell dependent it provides further evidence for a T cell anti-tumour immune response. This was clearly different to the three devils that did not show signs of remission as there was no indication of immune cell infiltration into the tumour. All devils that did not undergo remission had low to modest antibody levels, with no evidence for an increase after immunotherapy.

The analysis of MHC type and response to immunisation and immunotherapy is consistent with MHC incompatibilities inducing alloreactivity. The non-immunised control devil that did not respond to immunotherapy had a number of MHC ‘mismatches’ with the DFTD tumour cells. The failure of this devil to produce an immune response against live DFTD cells that had been treated with IFN-γ is consistent with the need for prior immunisation and highlights the complexity faced with developing a vaccine and/or immunotherapy. Cells exposed to IFN-γ, not only upregulate MHCI/II, but have also been found to upregulate expression of immune inhibitory molecules, such as B7-H1/PD-L1, in human and mouse tumour cell lines^24^.

Taken together, our results highlight the feasibility of developing a vaccine to counter the devastating Tasmanian devil facial tumour disease. The immune system of the Tasmanian devil is able to mount specific humoral and cellular responses to facilitate the eradication of established tumours. DFTD cells cultured in IFN-γ upregulate MHC-I and by incorporating preparations of these cells with adjuvants that target TLRs^25^ immune responses against the tumour cells are consistently produced. One potential complication could be the upregulation of PD-L1, which also occurs following IFN-γ exposure^26^. However, PD-L1 could also provide a useful target for immunotherapy, which has been successfully used for human cancers. An additional complication could be the discovery of a second transmissible cancer, DFT2^19^. A key challenge is to develop a strategy to protect against both DFTDs. The immunisations alone in this study did not completely prevent tumour engraftment, indicating that stronger protective immune responses will be required. Further studies are required to dissect both the immune and tumour suppressor mechanisms at play, and to find opportunities for blocking them, before we can develop effective immune control of DFTD.

## Methods

### Tasmanian devils and biological samples

The Tasmanian devils were housed in secured shelters in accordance with the guidelines of the Department of Primary Industries, Parks, Water and Environment of Tasmania (DPIPWE). All animal procedures were approved by the University of Tasmania Animal Ethics Committee under A009215, A0011436 and A0013685. All devils (6 females and 3 males) were adults with ages ranging from 5 to 7 years at commencement of experiments (Supplementary Table S5). All experimental methods were performed in accordance with the University of Tasmania guidelines.

Immunisation, blood collection, live cell challenge and therapy were performed under anaesthesia induced with 5% Isoflurane reducing to 3% via a mask (ISOTHESIA^®^ (Henry Schein, Northgate, Australia). 10 ml of blood was obtained from the jugular vein as previously described^27^. Up to 4 ml of blood was placed into clot activating tubes (Greiner Bio-one) for serum analysis and the remainder into lithium heparin anticoagulant tubes for cell analyses. Tumour biopsies were collected using sterile 4 mm disposable biopsy punches (Kai Medical). Biopsies were divided with a scalpel blade and half placed into 1 ml 10% neutral buffered formalin and half into 1 ml RNAlater.

### DFTD cell line culture conditions

The DFTD cell line C5065 (strain 3) was used for all experiments and was provided by A-M. Pearse and K. Swift of the DPIPWE. The cell line was maintained in RPMI 1640 culture medium (Life Technologies), supplemented with 10% heat inactivated foetal bovine serum (Bovogen), 1% GlutaMAX™ (Life technologies) and 1% Antibiotic-Antimycotic (Life Technologies) in a 35 °C humidified 5% CO2 incubator. When required, the cells were gently flushed from their culture surface using culture medium and pelleted by centrifugation at 500 g for 5 min. Cell viability counts were performed on an improved Neubauer chamber.

### Preparation of DFTD cells for immunisation and challenge

Four immunisation protocols (A-D) were evaluated. Complete details for dose, route, frequency of administration and vaccine composition for each immunisation protocol are indicated in Supplementary Table S6. Protocol D also included an adjuvant control devil that received four immunisations of the adjuvant components only (i.e. excluding DFTD cells).

MHC-I was upregulated in DFTD cells by treatment with either: (i) 10% cytokine rich conditioned medium (supernatant of Tasmanian devil MNC stimulated with 5 μg/ml Concanavalin A for 48 h) incubated for 48 h; (ii) Trichostatin A - TSA (Sigma-Aldrich) at 10 ng/ml in culture for 72 h; (iii) IFN-γ at 1/5,000 dilution in culture for 24 h; or (iv) 10% cytokine rich conditioned medium plus recombinant devil IFN- γ at 1/10,000 dilution in culture for 24 h.

The antigenic component of the vaccines included total protein extracted from DFTD cells that were heat-treated at 56 °C for one hour; DFTD cells inactivated by freeze/thawing cycles (10 quick cycles of freezing in liquid nitrogen and thawing in a 40 °C water bath); DFTD cells inactivated by radiation (two doses of 40 Gy of gamma radiation 24 h apart using a Varian Clinac 23-EX linear accelerator – Varian Medical Systems Inc.); or sonicated DFTD cells (four ultra-sonic cycles using 50% power using an ultrasonic cell disruptor – Misonix Inc.).

Adjuvants included ISCOMATRIX™ adjuvant (provided by CSL Ltd, Victoria, Australia) and the TLR agonists CpG oligonucleotide 1585 (CpG 1585, GeneWorks); CpG oligonucleotide 2395 (CpG 2395, GeneWorks) or poly(I:C) (poly I:C, Sigma-Aldrich).

Five immunised devils (TD1-My, TD2-Ga, TD3-Ty, TD4-Mm) were challenged with a single dose of 25,000 live DFTD cells (cell line C5065) resuspended in 0.25 ml of PBS injected subcutaneously into to the rump. Two immunised devils (TD6-Tp and TD7-Sy) and the non-immunised control (TD8-Mk) were challenged with 100,000 live DFTD cells in 1 ml of PBS into the left-hand side of the rump and 25,000 live DFTD cells into the right-hand side of the rump. The areas of injection were shaved for easier visualization and to mark the location. The devils were examined monthly under anaesthesia for evidence and measurement of tumours.

### Immunotherapy

A range of treatments were tested on the devils that developed tumours after the challenge. Therapies included administration of: (i) cytokine rich conditioned medium injected into the tumour; (ii) live cytokine-treated DFTD cells injected subcutaneously; (iii) cytokine-treated and then irradiated DFTD cells injected subcutaneously; and (iv) recombinant devil IFN-γ injected into the tumour. Details of the therapy protocols are indicated in Supplementary Table S7.

### Antibody responses analysis

Washed DFTD tumour cells (100 μl at 2 × 10^6^/ml diluted in washing buffer −0.5% BSA in PBS) were placed in wells of a round bottom 96 well plate on ice. Pre-immune and immune serum samples were diluted 1:50 with washing buffer, mixed with C5065 DFTD tumour cells and incubated on ice for 1 h. Cells were washed twice with washing buffer, and incubated with 50 μl of 10 μg/ml of a monoclonal mouse anti-devil IgG (provided by The Walter and Eliza Hall Institute)^8^ for 30 min, washed and incubated with 50 μl of 2 μg/ml Alexa Fluor 647 conjugated goat anti-mouse IgG antibody (Invitrogen) for 30 min. After washing, cells were resuspended in 200 μl of washing buffer containing 200 ng/ml of the cell viability dye 4’,6-Diamidino-2-Phenylindole, Dilactate (Sigma-Aldrich). Data acquisition was performed on a BD CANTO II flow cytometer (Becton Dickinson). The controls (DFTD cells labelled with the secondary and tertiary antibodies, but no devil serum) did not show background fluorescence. The median fluorescence intensity ratio (MFIR) was used to classify the antibody responses. The MFIR is the median fluorescence intensity (MFI) of DFTD cells labelled with immune serum divided by the MFI of DFTD cells labelled with pre-immune serum. The responses were considered: negative for MFIR <1.5 times the pre-immune response; low for MFIR of 1.5 to 2 times the pre-immune response; medium for MFIR of 2 to 4 times the pre-immune response and strong for MFIR >4 times the pre-immune response.

### Histology and immunohistochemistry

Standard haematoxylin and eosin (H&E) and immunohistochemical staining were performed on three-micrometre paraffin sections from tumour tissues fixed in 10% neutral buffered formalin as previously described^3^. Primary antibodies used for immunohistochemistry were as follows: Dako: anti-human CD3 (A0452; 1:300), anti-human HLA-DR - alpha chain (M0746; 1:40); Sigma-Aldrich: anti-human periaxin (HPA001868; 1:300). The Walter and Eliza Hall Institute provided the anti-devil CD4 (1:50) and anti-devil CD8 (1:100)^8^. Assessment of the biopsy samples and staining was performed by a Pathology Registrar (in human pathology). A light microscope (Olympus-BX50) coupled with a camera (Leica-DFC320) was used for visualization and acquisition of the images.

### MHC typing

All nine devils and the DFTD tumour cell line used in the study were genotyped at three classical MHC-I loci via allele cloning and sequencing. The genotyping protocol described previously^4^ was used with a few modifications. Briefly, genomic DNA was extracted from blood using the QIAamp DNA Blood Mini Kit (QIAGEN). MHC peptide-binding domains encoded by exon 2 and 3 of the genes were amplified with a pair of multilocus primers (forward: 5′-GTGTCCCCCCCTCCGTCTCAG-3′; reverse: 5′-GGGAATAATGGGGCAGGGGAGGT-3′) using the Platinum Taq DNA Polymerase High Fidelity Kit (Invitrogen). PCRs were performed on a Bio-Rad MJ Mini Personal Thermal Cycler with the following conditions: 100 °C hot lid; 94 °C initial denaturation for three minutes; 32 cycles of 94 °C denaturation for 30 seconds, 60 °C annealing for 30 seconds, and 72 °C extension for one minute; and 72 °C final extension for 10 minutes. PCR products were purified from agarose gel using QIAquick Gel Extraction Kit (QIAGEN) and cloned in a pGEM T Easy Vector (Promega A1360)/JM109 High Efficiency Competent Cells (Promega L1001) cloning system. Sixteen to twenty positive clones were picked for each sample and plasmids were extracted using DirectPrep 96 MiniPrep Kit (QIAGEN) on a QIAvac Multiwell vacuum manifold (QIAGEN). Purified plasmids were sequenced in both directions at the Australian Genome Research Facility. Two independent PCRs were performed for each individual and sequence variants were considered as real alleles only if they were found in more than one PCR amplification. Sequencing results were quality checked in Sequencher 4.1.4 (Gene Codes) and aligned with previously reported devil MHC alleles downloaded from GenBank in BioEdit^28^ and MEGA5^29^.

### Production of recombinant devil IFN-γ

A cDNA encoding full length devil IFN-γ (Ensembl reference ENSSHAG00000015036) bearing a 5′ Kozak sequence (GAAACC) and a C-terminal fusion to a Gly3His6 tag was synthesized by DNA2.0 (California, USA) and subcloned into pFastBac1 as a BamHI-NotI fragment. The sequence was confirmed by Sanger sequencing (Micromon, Monash University, VIC) before the construct was transformed into DH10MultiBac cells and the bacmid prepared as described previously^6^,^30^. Sf21 cells were transfected according to established protocols^30^, followed by two rounds of viral amplification to generate P3 virus. Sf21 cells (0.4 L) were cultured in 2.8 L Fernbach flasks in SF900-II SFM medium (Life Technologies) shaking at 90 rpm, 27 °C to a density of >2 × 10^6^ cells/ml before addition of P3 virus and continued incubation for 72 h. The volume of P3 virus per 10^6^ cells required for optimal protein yield was determined empirically. Cells were eliminated by centrifugation and the supernatant was subjected to tangential flow concentration and three cycles of buffer exchange into MT-PBS using a 10 kD molecular weight cut off ultrafilter. The concentrate was subjected to Ni^2+^ chromatography before elution in MT-PBS containing 180 mM NaCl and 250 mM imidazole pH 8, followed by concentration by centrifugal ultrafiltration and Superdex-75 gel filtration chromatography with elution in MT-PBS. Fractions confirmed to contain devil IFN-γ by SDS-PAGE were pooled, snap frozen in liquid nitrogen and stored at −80 °C until required.

## Additional Information

**How to cite this article**: Tovar, C. *et al*. Regression of devil facial tumour disease following immunotherapy in immunised Tasmanian devils. *Sci. Rep.* **7**, 43827; doi: 10.1038/srep43827 (2017).

**Publisher's note:** Springer Nature remains neutral with regard to jurisdictional claims in published maps and institutional affiliations.

## Supplementary Material

Supplementary Information

## Figures and Tables

**Table 1:** Summary of antibody and cellular responses to immunotherapy.

Imm. Protocol	Challenge and immunotherapy protocol	Tumour engraftment (palpable tumours)	Antibody response against DFDT cells^a^		Immune cell infiltration^b^				Tumour regression
			MHC-I (−)	MHC-I (+)	CD3^+^	CD8^+^	CD4^+^	MHC-II^+^	
**A**									
TD1-My	Challenge with live DFTD cells	37 days							
	Therapy: one dose of live conditioned medium-treated DFTD cells		++	+++	+++	+++	+	+++	Yes
	Therapy: 3 doses of conditioned medium intratumoural		NA	NA	+++	+++	+	++	
	Therapy: one dose of live conditioned medium-treated DFTD cells		+	+++	+++	+++	−	+++	
**B**									
TD2-Ga	Challenge with live DFTD cells	67 days	++	++					
	Therapy: one dose of irradiated IFN gamma-treated DFTD cells		++	++	+	+	+	+	No
	Therapy: one dose of IFN gamma intratumoural		NA	NA	+	+	+	-	
TD3-Ty	Challenge with live DFTD cells	67 days	−	−					
	Therapy: 3 doses of IFN gamma intratumoural		−	−	+	+	+	+	No
	Therapy: one dose of irradiated IFN gamma-treated DFTD cells		++	+	+	+	+	++	
**C**									
TD4-Mm	Challenge with live DFTD cells	No evidence of tumour development	++	+++					
TD5-Br	Euthanised before challenge								
**D**									
TD6-Tp	Challenge with live DFTD cells at the left and right hand side of the rump	80 days							Yes
	Therapy: live IFN gamma-treated DFTD cells		+	++	++	++	++	+++	
TD7-Sy	Challenge with live DFTD cells at the left and right hand side of the rump	110 days	++	++					Yes
	Therapy: live IFN gamma-treated DFTD cells		+++	+++	+++	++	+++	+++	
**Control**									
TD-9 Mk	Challenge with live DFTD cells at the left and right hand side of the rump	40 days							No
	Therapy: live IFN gamma-treated DFTD cells		−	−	+/-	+/−	+/−	+	

^a^Flow cytometry analysis. The median fluorescence intensity ratio (MFIR) was used to classify the antibody responses. The MFIR is the median fluorescence intensity (MFI) of DFTD cells labelled with immune serum divided by the MFI of DFTD cells labelled with pre-immune serum. The responses were considered.

− MFIR <1.5 times the pre-immune response.

+ MFIR 1.5 to 2 times the pre-immune response.

++ MFIR 2 to 4 times the pre-immune response.

+++ MFIR >4 times the pre-immune response.

^b^ Immune cell infiltration assessed by immunohistochemistry.

- No cells in the tumour or surrounding stroma.

+/− Few cells in the stroma but not infiltrating the tumour.

+ Occasional cells in the tumour.

++ Few scattered cells in the tumour.

+++ Large number of cells within the tumour. NA Not assessed.

**Table 2:** Comparison of immune responses in devils with regressed and non-regressed DFTD tumours.

Devil	Antibody response to immunisation		Immunotherapy	Antibody response following immunotherapy	Immune cell infiltration
	MHC-I^-^	MHC-I^+^			
**Tumour regression**					
TD1-My	Negative	Not assessed	Live MHC-I^+^ DFTD cells	Increased	Dense
(Protocol A)					
TD6-Tp	Medium	Medium			
(Protocol D)					Dense
TD7-Sy	Medium	Medium			
(Protocol D)					Dense
**No tumour regression**					
TD2-Ga	Low	Medium	Irradiated MHC-I^+^ DFTD cells	Did not change	
(Protocol B)					Sparse
TD3-Ty	Negative	Medium			
(Protocol B)					Sparse
TD8-Mk			Live MHC-I+ DFTD cells		
(Non-immunised control)					Sparse
