# Supplementary material for: Regression of devil facial tumour disease following immunotherapy in immunised Tasmanian devils
Source: Sci Rep. 2017 Mar 9;7:43827. doi: 10.1038/srep43827 (PMC5343465; doi:10.1038/srep43827)
Supplement: Supplementary Information [file srep43827-s1.pdf]

Regression of devil facial tumour disease following immunotherapy in immunised Tasmanian devils

Authors
Cesar Tovar[1^*]
Ruth J Pye[1^]
Alexandre Kreiss[1^]
Yuanyuan Cheng[2]
Gabriella K Brown[1,3]
Jocelyn Darby[1]
Roslyn C Malley[4,5]
Hannah V T Siddle[6]
Karsten Skjødt[7]
Jim Kaufman[8]
Anabel Silva[9]
Adriana Bazmorelli[9]
Anthony T Papenfuss[10]
Lynn M Corcoran[10,11]
James M Murphy[10,11]
Martin J Pearse[9]
Katherine Belov[2]
A Bruce Lyons[4]
Gregory M Woods[1,4*]

[1]Menzies Institute for Medical Research, University of Tasmania, Hobart, Tasmania 7000, Australia. [2]Faculty of Veterinary Science, University of Sydney, Sydney, New South Wales 2006, Australia. [3]Department of Health and Human Services, Hobart, Tasmania 7000, Australia. [4]School of Medicine, University of Tasmania, Hobart, Tasmania 7000, Australia. [5]Royal Hobart Hospital, Hobart, Tasmania 7000, Australia. [6]Centre for Biological Sciences, University of Southampton, Southampton SO17 1BJ, United Kingdom. [7]Department of Cancer and Inflammation, University of Southern Denmark, 5000 Odense C, Denmark. [8]Department of Pathology, University of Cambridge, Cambridge CB2 1QP, United Kingdom. [9]CSL Ltd., Bio21 Institute, Melbourne, Victoria 3010, Australia. [10]The Walter and Eliza Hall Institute of Medical Research, Parkville, Victoria 3052, Australia. [11]Department of Medical Biology, The University of Melbourne, Parkville, Victoria 3010, Australia.

^These authors contributed equally to this work.

*Correspondence and material requests should be addressed to G.M.W. (email: g.m.woods@utas.edu.au) or to C.T. (email: cdtovar@utas.edu.au).

Supplementary Information

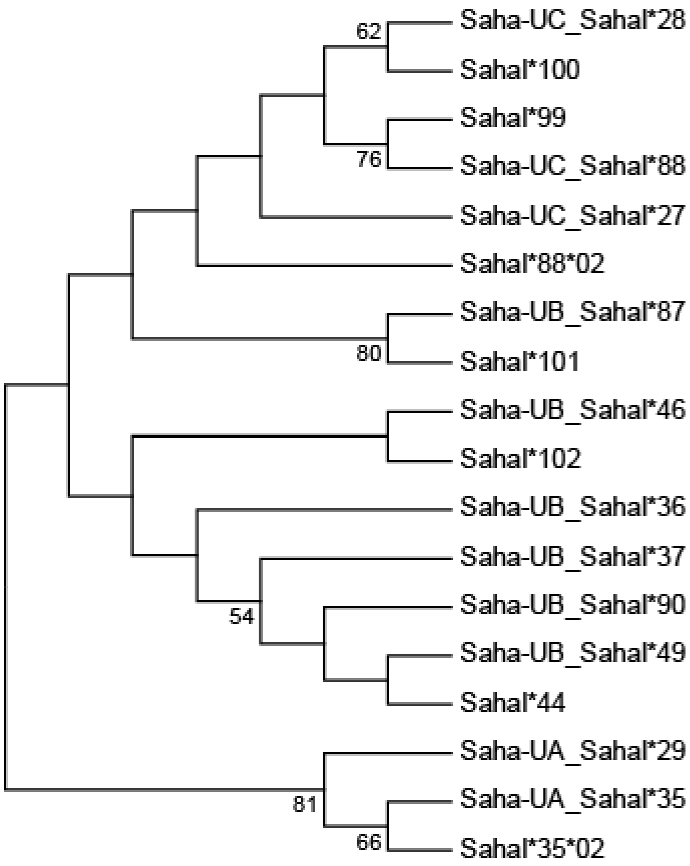

Supplementary Figure S1. Phylogenetic analysis of MHC I alleles (amino acid sequence of α1 and α2 domains) using the Neighbor-joining method. Bootstrap values lower than 50% are not shown. Gene names (Saha-UA, -UB, and -UC) are shown for alleles that have been previously assigned to loci based on BAC contig assembly and sequencing and genotype analysis (1, 2).

1. Cheng Y, Stuart A, Morris K, Taylor R, Siddle H, Deakin J, et al. Antigen-presenting genes and genomic copy number variations in the Tasmanian devil MHC. BMC Genomics. 2012;13:87.

2. Lane A, Cheng Y, Wright B, Hamede R, Levan L, Jones M, et al. New insights into the role of MHC diversity in devil facial tumour disease. PLoS One. 2012;7(6):e36955.

Supplementary Table S1. Summary of the immunisation, challenge and tumour development

Protocol	Year 1	Year 2	Year 3	Year 4	Year 5
**A**					
TD1-My	• • • * ↓ ◊ (37 days)				
**B**					
TD2-Ga		• • ↓ ◊ (67 days)			
TD3-Ty		• • ↓ ◊ (67 days)			
**C**					
TD4-Mm			• • • • *	* ↓	
TD5-Br			• • • • *		
**D**					
TD6-Tp				••• • * ↓	◊ (80 days)
TD7-Sy				••• • * ↓	◊ (110 days)
**Controls**					
Adjuvant control (TD9-Pl))				••• • *	
No-immunisation control (TD8-Mk)					↓ ◊ (40 days)

• Immunisation * Booster ↓ Challenge ◊ Tumour development (days after challenge)

Supplementary Table S2. Analysis of tumour morphology and immune cell infiltrate in the tumour growth phase

Immunisation protocol A	Immunisation protocol B		Immunisation protocol D		No-immunisation control
**TD1-My**	**TD2-Ga**	**TD3-Ty**	**TD6-Tp**	**TD7-Sy**	**TD8-Mk**
**Challenge 25,000 live DFTD cells**	**Challenge 25,000 live DFTD cells**	**Challenge 25,000 live DFTD cells**	**Challenge - 25,000 live DFTD cells at right hand side (RHS) of the rump + 100,000 live DFTD cells at the left hand side (LHS) of the rump**		**Challenge - 25,000 live DFTD cells RHS rump + 100,000 live DFTD cells LHS rump**
Biopsy 10 weeks after challenge<br>Tumour: Strong labelling for periaxin.The tumour incited a strong desmoplastic tissue response. No evidence of necrosis.<br><br>MHC-II: Few positive cells in the periphery of the tumour in association with T cells. Some occasional cells within the tumour.<br><br>T cells: No intratumoural T cells. Few cells in the periphery of the tumour, mostly CD8. There are no CD4 T cells.	Biopsy 12 weeks after challenge<br>Tumour: Strong labelling for periaxin. One area more packeted tumour architecture with periaxin staining only occurring in some of the cells. Small areas of tumour necrosis.<br><br>MHC-II: Cells with dendritic cell morphology are identified more densely at the edge of the tumour but also scattered throughout the tumour. No relationship to T cells. WIthin the dermis MHC-II postive cells are present.<br><br>T cells: Only occasional intratumoural CD3 cells. The number of CD8 cells correlates with the CD3 cell density. No CD4 cells are identified.	Biopsy 10 weeks after challenge<br>Tumour: The biopsy contains two fragments with similar architecture. Strong cytoplasmic staining for periaxin. Tumour cells with a high nuclear:cytoplasmic ratio. Tumour cells in single lines. Small amount of stroma separating lines of tumour cells.<br><br>MHC-II: Cells with a dendritic cell mophology can be seen in the epidermis, dermis and subcutaneous tissue. There are MHC-II cells on the periphery of the biopsy in association with the T cells. There are no intratumoural MHC-II cells.<br><br>T cells: Fragment 1: Small infiltrate of CD3 cells and small infiltrate of CD8 cells which correlates with CD3. Small infiltrate of CD4 (less than CD8).<br>Fragment 2: Occasional CD3 cells located near tumour cells. Occasional CD8 cells and very occasional CD4 cells located near tumour cells. The CD4 and CD8 cells seem to cluster in the same areas on the tumour edge.	Biopsy 12 weeks after challenge<br>Tumour: Single fragment of subcutaneous fat almost entirely replaced with spindle shaped tumour cells and desmoplastic stroma response. Variable tumour staining with periaxin ranging from weak to very strong.<br><br>MHC-II: Moderate number of MHC-II cells with occasional clusters in areas with lower numbers of periaxin positive tumour cells.<br><br>T cells: Generally small numbers of CD3 cells within the tumour with occasional clusters of CD3 cells that co-locate with the clusters of MHC-II cells.	Biopsy LHS tumour - 20 weeks after challenge<br>Tumour: 2 fragments. One tumour, one hair bearing skin and subcutaneous tissue containing a periaxin positive peripheral nerve. In the tumour fragment there is extensive tumour necrosis with viable tumour cells encircling intratumoural vessels. There is a desmoplastic tissue response. The tumour cells show variable nuclear periaxin staining. In some areas the majority of tumour cells are positive and elsewhere the majority negative.<br><br>MHC-II: Moderate dermal infiltrate of MHCII positive cells in dermis of non-tumour bearing skin. Small to large clusters of MHC-II cells in periphery of the tumour where the tumour is less periaxin positive. Few MHC-II cells in the areas where the tumour is most periaxin positive.<br><br>T cells: Few CD3 cells in periphery of tumour, correlating with the most dense areas of MHC-II cells. Very few intratumoral T cells.	Biopsy 10 weeks after challenge<br>Tumour: The specimen (T1) consists of a small biopsy of hair bearing skin and of three tumour containing fragments, two of which contain subcutaneous fat and muscle. There is a strong desmoplastic stromal response. A few intratumoural vessels are present. Periaxin positive cells are identified within the desmoplastic stroma with strong cytoplasmic staining and a plump spindled appearance.<br><br>MHC-II: Moderate numbers of MHC-II cells at the tumour edge. Small to moderate numbers of intertumoural MHC-II cells in all three fragments.<br><br>T cells: Small numbers of CD3 cells at the tumour edge. Very occasional intratumoural CD3 cells.
Biopsy 14 weeks after challenge<br>Tumour: Tumour has variable periaxin staining and appears encapsulated.<br><br>MHC-II: Moderate infiltrate of MHC-II cells around the tumour, blood vessels and surrounding connective tissue but only occasional positive cells within the tumour.<br><br>T cells: Moderate infiltrate of CD3 cells in the tumour. CD8 cells infiltrate correlates with CD3. No CD4 infiltrate.	**Therapy: Irradiated IFN gamma-treated DFTD cells 14 weeks after the challenge**<br>Biopsy one week after the therapy<br>Tumour: Strongly positive for periaxin, predominantly spindled cell populations. Necrosis is evident. Some tumour cells identified in vessels.<br><br>MHC-II: There are few MHC-II cells in the tumour fragment.<br><br>T cells: Few CD3 positive cells in this biopsy, largely in connective tissue areas. About 50% of the CD3 positive cells are CD8 and are predominantly in the connective tissue with only a few intratumoural CD8 cells. Very few CD4 cells.	**Therapy: IFN gamma intratumoural (3X weekly) 10 weeks after the challenge**<br>Biopsy one week after last therapy<br>Tumour: Strongly positive for periaxin. Pleomorphic, more densely packed tumour cells with little intervening stroma and a infiltrative margin. Small areas of tumour necrosis. Tumour present in lymphatics. Small vessels identified with in tumour.<br><br>MHC-II: Very occasional MHC-II positive cells within tumour.<br><br>T cells: CD3 cells present in small numbers. Infiltration in the periphery of the tumour mass within the connective tissue, but not in dense tumour tissue. CD8 cells present in same areas as CD3 represent approximately 50% of the cells. Very occasional CD4 cells.	Biopsy (T1- left hand side - LHS) 14 weeks after challenge<br>Tumour: 3 tissue fragments, one hair bearing skin, one muscle with haemorrhage and the largest fragment contains tumour. The tumour shows tumour angiogenesis with multiple small blood vessels and a small area of tumour necrosis. The tumour cells palisade around the tumour vessels with a few apoptotic cells most distant from the vessel. There is a strong desmoplastic stromal response. There is moderate, largely cytoplasmic periaxin staining in the tumour cells. Small numbers of cells with nuclear staining for periaxin are present within the small, muscle containing fragment.<br><br>MHC-II: Variable infiltrate with small-moderate numbers of MHC-II cells within the tumour, particularly in the spindled desmoplastic stroma and some in clusters next to apoptotic cells. Moderate numbers of MHC-II cells within the smaller fragment containing muscle.<br><br>T cells: Small numbers of CD3 cells are present throughout the fibrous connective tissue in the fragment containing muscle. Small numbers of CD3 cells are present throughout the tumour containing fragment but most notably in the desmoplastic stroma and adjacent to tumour vessels.	**Therapy MHC-I+ live DFTD cells 20 weeks after challenge**<br>Biopsy (left hand side tumour -LHS) - 2 weeks after therapy<br>Tumour: 2 fragments. One tumour, one hair bearing skin and subcutaneous tissue containing a periaxin positive peripheral nerve. Majority of tumour cells have nuclear staining periaxin. Tumour cells are closely packed and there is a strong desmoplastic response. Small area of tumour necrosis is present.<br><br>MHC-II: Moderate numbers of MHC-II cells in the periphery of tumour, particularly in the desmoplastic stroma. Moderate numbers of small MHC-II cell clusters also present intratumorally. Most are in association with T cells.<br><br>T cells: Moderate numbers of CD3 T cells in periphery of the tumour. Small numbers within the tumour.	**Therapy MHC-I+ live DFTD cells 10 weeks after challenge**<br>Biopsy 2 weeks after therapy<br>Tumour: The specimen (T1) consists of a fragment of hair bearing skin and a fragment of tumour. The tumour shows tumour angiogenesis with tumour cells palisading around intratumoural vessels with intervening necrosis. There is a well developed desmoplastic stromal response and the tumour cells have moderate cytoplasmic periaxin staining.<br><br>MHC-II: Moderate numbers of MHC-II cells around the edge of the the tumour with very occasional MHC-II cells within the tumour.<br><br>T cells: Small numbers of CD3 cells at the edge of the tumour paralleling the MHC-II cell infiltrate. No intratumoural CD3 cells.
Biopsy 17 weeks after challenge<br>Tumour: Areas of tumour necrosis and tumour angiogenesis. Viable tumour around new vessels.<br><br>MHC-II: MHC-II cells on the periphery of the tumour and in clusters in surrounding large blood vessels within the tumour. There are scattered intratumoural MHC-II cells.<br><br>T cells: CD3 looks like some are coming from the blood vessels. Mild infiltrate around the blood vessels and in the periphery. The CD3 cells are largely CD8 as there are no positive CD4 cells.	**Therapy: IFN gamma intratumoural 16 weeks after the challenge**<br>Biopsy one week after therapy<br>Tumour: Strongly periaxin postive tumour identified in the subcutaneous fat. Some weaker periaxin stained cells. No tumour angiogenesis.<br><br>MHC-II: Occasional MHC-II cells identified the tumour and few identified in the dermis.<br><br>T cells: Occasional intratumoural CD3 cells and a few around blood vessels in the subcutaneous fat. About 25% of CD3 cells are CD4 and 75% CD8.	**Therapy: Immunisation with irradiated IFN gamma treated DFTD cells 5 weeks after last therapy**<br>Biopsy 4 days later<br>Tumour: Biopsy of the tumour site after the tumour dislodgement shows reactive granulation tissue including activated fibroblasts, with prominent nucleoli, throughout the adjacent adipose tissue. No definite tumour cells identified by periaxin expression, but atypical cells identified. Necrotic tumour tissue was identified on the edge of the biopsy.<br><br>MHC-II: Large dendritic cells seen on the periphery of the tumour necrosis in the area of the CD3/CD8 infiltrate. Mild MHC-II cell infiltrate throughout the adipose tissue and dermis.<br><br>T cells: Scattered CD3 cells throughout the biopsy but most dense on the deep edge of the biopsy adjacent necrotic tissue. Most of the CD3 cells on the periphery of the necrotic tumour are CD8. Occasional CD4 cells are identified.	**Therapy MHC-I+ live DFTD cells 14 weeks after challenge**<br>Biopsy (T1 LHS) 2 weeks after therapy<br>Tumour: 3 tissue fragments, one ulcerated hair bearing skin with underlying granulation tissue and dense fibrous connective tissue in which small numbers of moderate peraxin positive cells can be seen adjacent to the surgical margin; one small fibrotic piece of tissue containing small nodules of weak periaxin cells; and a larger fragment comprised of largely dense connective tissue and small area of moderate periaxin positive tumour cells with a central area of necrosis. Tumour angiogenesis and desmoplastic stromal response are prominent features.<br><br>MHC-II: Moderate numbers of MHC-II cells within the tumour bearing fragments particularly in the desmoplastic stromal response but most dense surrounding (but not within) the periaxin containing foci and the dermis underlying the ulcerated skin.<br><br>T cells: Moderate numbers of CD3 cells in all fragments. The infiltrate paralells the MHC-II cell infiltrate being largely in the desmoplastic stroma and immediately adjacent the periaxin positive cells. Occasional CD3 cells are identified within the tumour foci. Approximately 80% of tumour cells are CD8 the remainder being CD4.	Biopsy 4 weeks after therapy<br>Tumour: Three tumours sampled right hand side (RHS), middle and left hand side (LHS). All fragments have variable amounts of tumour infiltrating subcutaneous fat. RHS and middletumour inciting a strong desmoplastic response. The LHS tumour shows prominent intratumoural vessels which the viable tumour cells encircle. Intervening areas show tumour necrosis or replacement with desmoplastic connective tissue. All biopsies include hairbearing skin. In all tumours there is variable periaxin staining with many tumour cells completely negative.<br><br>MHC-II: Large numbers of MHC-II cells on the peripheral tumour cell cuff with smaller numbers within the tumour. MHC-II cells are in association with T cells.<br><br>T cells: Large numbers of CD3 cells that follow the same pattern of MHC-II cell infiltration in all tumours. There are less CD3 cells than MHC-II cells. There are slightly more CD8 than CD4 cells in RHS tumour; more CD4 cells than CD8 cells in the middle tumour and LHS tumour.	Biopsy (autopsy) 4 weeks after therapy<br>Tumour: 3 biopsies taken RHS, interscapular and LHS. RHS consists of a tumour with one edge appearing encapsulated. There is a strong desmoplastic response with numerous spindled cells. There are moderate numbers of intratumoral blood vessels and areas of acellular connective tissue which may represent scarring following necrosis.The tumour cells are cytoplasmic periaxin (moderate labelling) and the spindle cells are periaxin negative. The interscapular tumour fragment appears similar to the RHS tumour in morphology and periaxin staining, however the tumour also has necrotic areas and infiltrates subcutaneous fat. The LHS tumour appears similar to the RHS and scapular tumours, however it has well developed islands of tumour palisading around medium to large sized intratumoural blood vessels and extensive necrosis between these islands of tumour cells. Small round packets of tumour cells may represent extensive lymphovascular permeation of tumour cells. The tumour cells show moderate to strong periaxin staining.<br><br>MHC-II: All tumours show similar MHC-II infiltrate. A few foci of MHC-II cells appear in moderate numbers on the periphery of the tumour with only small numbers of intratumoural MHC-II cells.<br><br>T cells: All tumours show a similar CD3 cell infiltrate. Very small numbers of CD3 cells on the periphery of the tumour. Very occasional intratumoral CD3 cells. The CD3 cells are in approximately equal numbers of CD4 and CD8 cells.
**Therapy: Live, conditioned medium-treated DFTD cells 20 weeks after the challenge**<br>Biopsy one week after the therapy<br>Tumour: Periaxin positive cells present only in the peripheral infiltrative border of the tumour. There is a variable pattern of periaxin staining within the connective tissue.<br><br>MHC-II: Very strong infiltrate of MHC-II cells within the tumour. MHC-II cells with dendritic morphology are present in the subcutaneous tissue.<br><br>T cells: Very strong infiltrate of CD3 throughout the tumour. Most of the CD3 cells are CD8 cells. Occasional CD4 cells.	Died of an unrelated cause 3 weeks after last therapy<br>Post-mortem biopsy<br>Tumour: Strong evidence of tumour vascularization with very dense large vessels within the tumours. Serpiginous bands of necrosis between the vessels. Tumour appears encapsulated but tongues of tumour tissue extend beyond the capsule. One fragment contains tumour cells with a nested/packed tumour cells with only occasional cells staining for periaxin.<br><br>MHC-II: MHC-II cells present in granulation tissue and in the connective tissue bands that are away from the tumour. Only occasional MHC-II cells within tumour cell nests.<br><br>T cells: Very few CD3 cells. Higher density adjacent to tumour capsule, near blood vessels. Very few in the tumour itself. Where there is granulation tissue there is a high density of CD3 cells, where there are equal numbers of CD8 and CD4 cells. Very few intratumoural CD8 and CD4 even around the large intratumoural vessels.	Biopsy 1 week after therapy<br>Tumour: On deep edge of the biopsy, strong periaxin stained tumour and peripheral nerves were identified. Desmoplastic tumour stroma response and granulation tissue adjacent to tumour.<br><br>MHC-II: Large infiltrate of MHC-II cells within the tumour areas and elsewhere including the dermis and epidermis.<br><br>T cells: Dense CD3 cell infiltrate around small pockets of tumour tissue but also in areas that are periaxin negative and in the upper dermis. CD8 cells correlate with density of CD3 cells. CD4 cells correlate with the density of CD3 cellsbut are in smaller numbers than the CD8 cells.	Biopsy (T1-LHS) 4 weeks after therapy<br>Tumour: The biopsy consists of four fragments of tissue. One fragment contains skin and underlying subcutaneous tissue including muscle. 2 small fragments contain fibrous tissue and fat. The largest fragment contains a focus of weakly periaxin tumour adjacent to subcutaneous fat and within a densely cellular desmoplastic tissue response. Weakly periaxin positive cells are identified within a large vessel distant to the tumour focus within the same fragment.<br><br>MHC-II: Large numbers of MHC-II cells infiltrate the desmoplastic stroma in the tumour bearing fragment (most marked adjacent to the tumour) as well as in the smaller non-skin bearing fragments. Small numbers of MHC-II cells can be seen within the periaxin tumour.<br><br>T cells: Moderate numbers of CD3 cells are seen within the tumour bearing fragment. Their density parallels that of the MHC-II cells being largely in the desmoplastic stroma with only a few present within the tumour. The non-tumour bearing fragments also contain small-moderate numbers of CD3 cells. There are approximately equal numbers of CD4 and CD8 cells.	Biopsy 6 weeks after therapy<br>Tumour: 2 biopsies. The RHS biopsy contains a section of hair bearing skin and 2 fragments of subcutaneous tissues. No tumour mass is identifed. No periaxin positive cells are identified in one fragment. In the other larger fragment has areas of fibrosis containing scattered strong periaxin positive cells. In the biopsy of the interscapular tumour there are two fragments of tissue, one hair bearing skin, the other containing tumour with small areas of necrosis. The tumour cells are more spindle shaped and contain many slit like small blood vessels. The surrounding tissue shows dense fibrosis. The tumour cells show variable periaxin staining, some with both nuclear and cytoplasmic staining.<br><br>MHC-II: In the RHS biopsy there are clusters of MHC-II cells in the dermis and subcutaneous tissues. In the RHS larger fragment some of the periaxin positive cells appear to correlate with MHC-II positive cells. In the interscapular tumour section there are large numbers of MHC-II postive cells surrounding the tumour, within the dense fibrosis. There are few MHC-II cells within the tumour.<br><br>T cells: In the RHS biopsy there are large numbers of CD3 cells infiltrating the tissue fragment with periaxin positive cells and smaller numbers in the smaller tissue fragment. There are approximately equal numbers of CD4 and CD8 cells in both fragments. In theinterscapular tumour biopsy moderate to large numbers of CD3 cells are surrounding the tumour, some infiltrating into the outer tumour border, correlating with the areas of MHC-II cells. There are slightly more CD8 cells than CD4 cells.	
**Therapy: Conditioned medium intratumoural (2X) in Week 23**<br>Biopsy one week after the therapy<br>Tumour: Only a few periaxin positive cells amoungst reactive fibroblasts. On the edge of the biopsy there are marked numbers of apoptotic cells.<br><br>MHC-II: Large infiltrate of MHC-II positive cells throughout the biopsy but not as frequent in the periaxin positive areas.<br><br>T cells: Large infiltrate of CD3 cells in tumour and areas containing apoptotic cells. Most CD3 cells are CD8 cells with only occasional CD4 cells.		Biopsy 3 weeks after therapy<br>Tumour: Periaxin- nests of tumour cells not stained well but individual cells even some with DC morphology are staining with periaxin.<br><br>MHC-II: Moderate infiltrate of MHC-II cells, particularly where the CD3 population lies and the border between the subcutaneous tissue, the tumour and desmoplastic stroma. Very few within the tumour mass.<br><br>T cells: Most CD3 cells are in the connective tissue around the tumour nests in the subcutaneous fat. Very few infiltrating nests of tumour itself. The CD3 infiltrate is composed mainly of CD8, cells particularly at the dermis/subcutaneous tissue. About 25% of CD3 are CD4.	10 weeks after the therapy Biopsy (LHS tumour)<br>Tumour: The biopsy consists of a large and small tissue fragment containing well circumscribed hyalinised relatively acellular dense connective tissue with very small numbers of cells around large blood vessels with adjacent fat and more cellular connective tissue. Occasional small strong periaxin postitive cells are seen largely in connective tissue adjacent to the acellular connective tissue and these are not thought to be tumour cells. A periaxin positive peripheral nerve is identified. This appears to represent mature scar tissue.<br><br>MHC-II: Moderate to large numbers of MHC-II cells singly and in clusters are seen adjacent to the acellular hylanised dense connective tissue with few MHC-II cells seen within the hyalinised connective tissue.<br><br>T cells: Moderate numbers of CD3 cells parallel the MHC-II cell infiltrate. There are few CD3 cells within the acellular hyalinised connective tissue. There are approximately 60% CD8 cells and 40% are CD4 cells.	Biopsy 8 weeks after the therapy<br>Tumour: Two biopsies taken labelled RHS T1 and interscapular. The RHS biopsy shows subcutaneous fat with marked areas of fibrosis with a single cell infiltrate of variable density. No definite tumour mass is identified, however small numbers of cells have nuclear strong staining for periaxin. The biopsy labelled interscapular consists of hair bearing skin with an area of dense fibrosis within the subcutaneous fat. Within the fibrotic area there are dense infiltrates of cells, small numbers of which are strongly-periaxin positive.<br><br>MHC-II: In the tumour labelled RHST1 there are large numbers of MHC-II infiltrating the entire specimen with small groups present around blood vessels. MHC-II cells are the predominant cell type in the cellular areas within the subcutaneous fat in the interscapular biopsy.<br><br>T cells: In the tumour labelled RHST1 there are large numbers of CD3 cells infiltrating the entire specimen with small groups present around blood vessels in association with MHC-II cells. Approximately 70% of these cells are CD4 and 30% are CD8. In the interscapular tumour, within the cellular areas within the fibrotic tissue in the subcutaneous fat moderate numbers of CD3 cells are present. These cells are predominantly CD4 with about 20% being CD8. There are marked numbers of CD4 and CD8 cells in the surrounding subcutaneous fat.	
**Therapy: Conditioned medium intratumoural (1X) in Week 24, then 1 week later treatment with live, conditioned medium-treated DFTD cells**<br>Biopsy one week after the therapy (Tumour regression)<br>Tumour: No periaxin staining.<br><br>MHC-II: MHC-II cells throughout biopsy and some small cell clusters are present.<br><br>T cells: The MHC-II infiltrate co-localises with a strong CD3 infiltrate. CD3 are mostly CD8. There are no CD4 cells identified.		Biopsy 5 weeks after therapy<br>Tumour: Four tumour samples differing in tumour morphology and periaxin staining. Some infiltrative spindled-large strongly periaxin positive tumour cells with strong desmoplastic stromal response. Some fragments show nests of malignant cells with no periaxin staining. Within these nests there are more dendritic-like, spindled periaxin positive tumour cells.<br><br>MHC-II: Cells seen in the periphery of the highly periaxin positive spindle tumour cells but not within tumour itself. MHC-II positive cells infiltrate the nests of periaxin (-) tumour cells. This implies this clone is able to infiltrate. The CD3 cells, mainly CD8, are still not gaining access to the nests of tumours in this site as they are mainly in connective tissue strands. The CD3 and MHC-II don't seem to meet.<br><br>T cells: In all tumour morphological types there is a moderate infiltrate of CD3 cells. CD8 positive cells correlated with CD3 cell infiltrate. CD4 cells were more prevalent in the periaxin positive tumour areas than elsewhere.		Biopsy (interscapular tumour) 10 weeks after the therapy<br>Tumour: One interscapular biopsy consists of hair bearing skin with underlying subcutaneous tissues. There is an area of cellular dense fibrotic tissue, organising granulation tissue with multiple blood vessels and on the periphery of the biopsy small groups of cells containing marked numbers of apoptotic cells. In these areas the cells are strongly stained with periaxin.<br><br>MHC-II: Within the cellular area of dense fibrosis there are large numbers of MHC-II cells. Large clusters of MHC-II cells are present within the areas periaxin positive tumour, and immediately adjacent to it.<br><br>T cells: CD3 cells are present in large numbers at the edge of the area of fibrosis and in moderate numbers within the area of fibrosis and in the areas of tumour and MHC-II cells. Approximately 70% are CD4 cells the remaining are CD8 cells.	

Supplementary Table S3. Genotypes of the nine devils and DFTD tumour cell line

Protocol	Animal ID	MHC I alleles[c] (SahaI*)					
		Saha-UA[a]		*Saha-UB*		*Saha-UC*	
A	TD1-My	-	-	101	101	88	88
B	TD2-Ga	-	-	87	87	88	88
	TD3-Ty	-	-	49	101	27	88
C	TD4-Mm	29	-	101	102	28	88
	TD5-Br	35:02[b]	-	46	101	28	88
D	TD6-Tp	29	29	36	37	27	100
	TD7-Sy	29	29	36	37	27	27
Control	TD9-Pl	35	-	44	101	88:02[b]	99
	TD8-Mk	29	-	87	102	28	88
	DFTD	35	35	46	90	28	28

[a] Dashes indicate Saha-UA pseudogene.

[b] Alleles SahaI*35:02 and SahaI*88:02 have the same α1 domains as alleles SahaI*35 and SahaI*88, respectively, but different α2 domains.

[c] Newly identified alleles were assigned to gene UA, UB or UC based on their phylogenetic relationship with previously reported variants (Figure S1). For example, SahaI*101 was assigned to UB as it clustered with UB allele SahaI*87 with 80% support. SahaI*102 was assigned to UB because a) it clustered with UB alleles (with low support though); b) the two animals containing this allele (TD4-Mm and TD8-Mk) had two confirmed UC alleles (SahaI*28 and 88) and a single copy of UA gene (SahaI*29), which excludes the possibility of SahaI*102 belonging to UA or UC.

Supplementary Table S4. Pair-wise sequence similarity among MHC I alleles

aa\nt[a]	SahaI *29	SahaI *35	SahaI* 35*02	SahaI *87	SahaI *101	SahaI *46	SahaI *102	SahaI *36	SahaI *37	SahaI *90	SahaI *49	SahaI *44	SahaI *28	SahaI *100	SahaI *99	SahaI *88	SahaI* 88*02	SahaI *27
SahaI*29	ID	0.995	0.998	0.97	0.967	0.986	0.975	0.99	0.985	0.98	0.977	0.98	0.978	0.98	0.969	0.972	0.98	0.977
SahaI*35	0.983	ID	0.996	0.969	0.962	0.988	0.977	0.995	0.99	0.982	0.982	0.985	0.973	0.975	0.964	0.967	0.975	0.972
SahaI*35*02	0.994	0.989	ID	0.972	0.965	0.985	0.973	0.991	0.986	0.978	0.978	0.982	0.977	0.978	0.967	0.97	0.978	0.975
SahaI*87	0.913	0.93	0.919	ID	0.982	0.96	0.954	0.97	0.969	0.97	0.964	0.973	0.96	0.962	0.967	0.964	0.969	0.962
SahaI*101	0.919	0.924	0.913	0.956	ID	0.964	0.972	0.964	0.965	0.959	0.959	0.969	0.978	0.98	0.985	0.982	0.986	0.98
SahaI*46	0.967	0.951	0.962	0.892	0.908	ID	0.988	0.983	0.988	0.986	0.986	0.983	0.975	0.977	0.965	0.969	0.977	0.977
SahaI*102	0.94	0.924	0.935	0.881	0.924	0.973	ID	0.978	0.983	0.983	0.983	0.98	0.986	0.985	0.973	0.977	0.985	0.988
SahaI*36	0.967	0.962	0.973	0.913	0.903	0.956	0.946	ID	0.995	0.986	0.986	0.99	0.975	0.977	0.965	0.969	0.977	0.973
SahaI*37	0.951	0.946	0.956	0.908	0.897	0.973	0.962	0.983	ID	0.988	0.991	0.995	0.977	0.978	0.967	0.97	0.978	0.978
SahaI*90	0.94	0.924	0.935	0.919	0.892	0.962	0.956	0.962	0.978	ID	0.99	0.986	0.97	0.972	0.96	0.964	0.972	0.972
SahaI*49	0.94	0.935	0.946	0.908	0.892	0.962	0.956	0.973	0.989	0.978	ID	0.99	0.973	0.975	0.964	0.967	0.972	0.975
SahaI*44	0.935	0.93	0.94	0.924	0.908	0.956	0.951	0.967	0.983	0.973	0.983	ID	0.973	0.975	0.97	0.967	0.975	0.975
SahaI*28	0.946	0.94	0.94	0.897	0.94	0.935	0.962	0.93	0.924	0.919	0.93	0.913	ID	0.998	0.986	0.99	0.988	0.995
SahaI*100	0.951	0.946	0.946	0.903	0.946	0.94	0.956	0.935	0.93	0.924	0.935	0.919	0.994	ID	0.988	0.991	0.99	0.993
SahaI*99	0.924	0.919	0.919	0.919	0.962	0.913	0.93	0.908	0.903	0.897	0.908	0.913	0.967	0.973	ID	0.996	0.988	0.985
SahaI*88	0.935	0.93	0.93	0.908	0.951	0.924	0.94	0.919	0.913	0.908	0.919	0.903	0.978	0.983	0.989	ID	0.991	0.988
SahaI*88*02	0.956	0.94	0.951	0.919	0.962	0.946	0.962	0.94	0.935	0.93	0.93	0.924	0.967	0.973	0.967	0.978	ID	0.99
SahaI*27	0.94	0.935	0.935	0.903	0.946	0.94	0.967	0.924	0.93	0.924	0.935	0.919	0.983	0.978	0.962	0.973	0.973	ID

[a] Above diagonal: nucleotide sequence identity; below diagonal: amino acid sequence identity

Supplementary Table S5. Sex, age and origin of the Tasmanian devils

Devil	Sex	Age at the time of experiments	Origin	Microchip number
TD1-My	Female	5 years	Wild born	175995
TD2-Ga	Female	6 years	Wild born	184165
TD3-Ty	Female	6 years	Wild born	161336
TD4-Mm	Female	6 years	Wild born	249252
TD5-Br	Female	7 years	Wild born	072697
TD6-Tp	Male	6 years	Captive born	781441
TD7-Sy	Female	7 years	Captive born	807727
TD8-Mk	Male	5 years	Captive born	210992
TD9-Pl	Male	5 years	Wild born	064640

Supplementary Table S6. Immunisation protocol

Immunisation protocol	Dose	Route	Frequency	Vaccine composition		Serum samples
				DFTD antigen	Adjuvant	
**A**						
TD1-My	3 doses	Subcutaneous, rump	Monthly	Two injections per dose of 200µg of DFTD protein extracted from heat-treated cells at 56°C for 1 h.	25 µl ISCOMATRIX	Weekly
	Booster	Subcutaneous, rump	6 months after last immunisation	One injection of 1000 µg of DFTD protein.	50 µl ISCOMATRIX	Weekly
**B**						
TD2-Ga	2 doses	Subcutaneous, rump	Monthly	One injection per dose of DFTD cells pre-treated with 10 ng/ml trichostatin A (TSA), incubated for 48 h. Immunised with total 2x10^6 to 1.5x10^7 cells (in 1000 µl), frozen in liquid nitrogen and thawed in 40°C water bath, ten cycles.	28 µl of ISCOMATRIX	Weekly
TD3-Ty	2 doses	Subcutaneous, rump	Monthly	One injection per dose of DFTD cells pre-treated with 10% conditioned medium, incubated for 48 h. Immunised with total 2.4x10^6 to 2x10^{7}cells (in 1000 µl), frozen in liquid nitrogen and thawed in 40°C water bath, ten cycles.	28 µl of ISCOMATRIX	Weekly
**C**						
TD4-Mm & TD5-Br	2 doses	Subcutaneous, interscapular	Monthly	One injection per dose of ~ 3x107 sonicated DFTD cells pre-treated with 10% conditioned medium plus IFN gamma 1/10,000, incubated for 24 h.	50 µl of ISCOMATRIX,<br>50 µg of CpG 1585<br>50 µg of CpG 2395<br>100 µl of poly I:C (1 mg/ml)	Fortnightly
	2 doses	Subcutaneous, interscapular	Monthly	Two injections per dose:		Fortnightly
				One injection of ~ 1x10^6 DFTD cells treated with 10% conditioned medium plus IFN gamma (1/10,000), incubated for 24 h then irradiated (2X 40 Gy, 24 h apart) and administered the following day.	Alone (no adjuvants)	
				One injection of ~ 1x10^6 DFTD cells treated with 10% conditioned medium plus IFN gamma (1/10,000), incubated for 24 h then irradiated (2X 40 Gy, 24 h apart) and administered the following day.	50 µl of ISCOMATRIX,<br>50 µg of CpG 1585<br>50 µg of CpG 2395<br>100 µl of poly I:C (1 mg/ml)	
	Booster 1 TD4 and TD5	Subcutaneous, interscapular	4 months after last immunisation	Two injections per dose as previous immunisation.	As previous immunisation	Fortnightly
	Booster 2 TD4	Subcutaneous, interscapular	7 months after last booster	One injection of ~ 2x10^6 DFTD cells treated with 10% conditioned medium plus IFN gamma (1/10,000), incubated for 24 h then irradiated (2X 40 Gy, 24 h apart) and administered the following day.	50 µl of ISCOMATRIX,<br>50 µg of CpG 1585<br>50 µg of CpG 2395<br>100 µl of poly I:C (1 mg/ml)	Fortnightly
**D**						
TD6-Tp & TD7-Sy	3 doses	Subcutaneous, interscapular	Fortnightly	One injection per dose of ~ 2x10^6 DFTD cells treated IFN gamma (1/5,000), incubated for 24 h then irradiated (2X 40 Gy, 24 h apart). Cells frozen in freezing medium, defrosted and washed in PBS before injection.	50 µl of ISCOMATRIX,<br>50 µg of CpG 1585<br>50 µg of CpG 2395<br>100 µl of poly I:C (1 mg/ml)	Fortnightly
	1 dose	Subcutaneous, interscapular	One month later	One injection of ~ 2x10^7 sonicated DFTD cells pre-treated with IFN gamma 1/5,000 for 24 h.	As previous immunisation	Fortnightly
	Booster	Subcutaneous, interscapular	6 months after last immunisation	One injection per dose of ~ 2x10^6 DFTD cells treated IFN gamma (1/5,000), incubated for 24 h then irradiated (2X 40 Gy, 24 h apart). Cells frozen in freezing medium, defrosted and washed in PBS before injection.	As previous immunisation	Fortnightly

Supplementary Table S7. Challenge and immunotherapy protocol

Protocol	Challenge and immunotherapy protocol	
**A**		
TD1-My	Challenge	25,000 live DFTD cells in 250 µl of PBS in one site on the rump.
	Therapy: one dose of $4x10^6$ live treated DFTD cells subcutaneously, 4 weeks after last treatment.	DFTD cells treated with 10% conditioned medium for 72 h. $1.3x10^7$ cells in 1,000 µl of PBS injected between shoulder blades.
	Therapy: three weekly doses of conditioned medium intratumoural, 2 weeks after last treatment.	4,000 µl of conditioned medium (medium prepared from devil mononuclear cells using 10% devil plasma and 10 µg/ml of Concanavalin A).
	Therapy: one dose of $1.5x10^7$ live treated DFTD cells subcutaneously, 4 weeks after last treatment.	DFTD cells treated with 2.5% conditioned medium for 48 h. $6.5x10^7$ cells in 1,000 µl of PBS plus 250 µl of conditioned medium injected between shoulder blades.
**B**		
TD2-Ga	Challenge	25,000 live DFTD cells in 1,000µl of PBS in one site on the rump.
	Therapy: one dose of treated and irradiated DFTD cells subcutaneously, 14 weeks after challenge.	DFTD cells treated with 1/50,000 IFN gamma for 72 h and irradiated with 40 Gy. $2x10^7$ cells plus 1/50,000 IFN gamma in in 1,000 µl of PBS, injected on the rump.
	Therapy: one dose of IFN gamma intratumoural, 2 weeks after last treatment.	3,000 µl of 1/10,000 IFN gamma in PBS.
TD3-Ty	Challenge	25,000 live DFTD cells in 1,000 µl of PBS in one site on the rump.
	Therapy: three weekly doses of IFN gamma intratumoural, 10 weeks after challenge.	2,000 µl of 1/10,000 IFN gamma in PBS.
	Therapy: one dose of treated and irradiated DFTD cells subcutaneously, 2 weeks after the last treatment.	DFTD cells treated with 1/50,000 IFN gamma for 72 h and irradiated with 40 Gy. $2x10^7$ cells plus 1/50,000 IFN gamma in 1,000 µl of PBS, injected on the rump.
**C**		
TD4-Mm	Challenge	25,000 live DFTD cells in 250 µl of PBS in one site on the rump.
	Did not develop a tumour	
TD5-Br	Euthanised before challenge	
**D**		
TD6-Tp	Challenge	100,000 live DFTD cells in 1,000 µl of PBS in left hand-side of rump plus 25,000 live DFTD cells in 250 µl of PBS in right hand-side of rump.
	Therapy: one dose of $8x10^6$ live treated DFTD cells subcutaneously, 3.5 months after challenge.	DFTD cells treated with 10% conditioned medium plus 1/10,000 IFN gamma for 24 h; interscapular injection.
TD7-Sy	Challenge	100,000 live DFTD cells in 1,000 µl of PBS in left hand-side of rump plus 25,000 live DFTD cells in 250 µl of PBS in right hand-side of rump.
	Therapy: one dose of $8x10^6$ live treated DFTD cells subcutaneously, 5 months after challenge.	DFTD cells treated with 10% conditioned medium plus 1/10,000 IFN gamma for 24 h; interscapular injection.
**Control**		
TD8-Mk	Challenge	100,000 live DFTD cells in 1,000 µl of PBS in left hand-side of rump plus 25,000 live DFTD cells in 250 µl of PBS in right hand-side of rump.
	Therapy: one dose of $8x10^6$ live treated DFTD cells subcutaneously, 10 weeks after challenge.	DFTD cells treated with 10% conditioned medium plus 1/10,000 IFN gamma for 24 h; interscapular injection.
